# Simulating Polymerization by Boltzmann Inversion Force Field Approach and Dynamical Nonequilibrium Reactive Molecular Dynamics

**DOI:** 10.3390/polym14214529

**Published:** 2022-10-26

**Authors:** Michele Monteferrante, Sauro Succi, Dario Pisignano, Marco Lauricella

**Affiliations:** 1 Istituto per le Applicazioni del Calcolo CNR, Via dei Taurini 19, 00185 Rome, Italy; 2 Center for Life Nano Science@La Sapienza, Istituto Italiano di Tecnologia, Viale Regina Elena, 291, 00161 Roma, Italy; 3 Dipartimento di Fisica, Università di Pisa, Largo B. Pontecorvo 3, 56127 Pisa, Italy; 4 NEST, Istituto Nanoscienze-CNR, Piazza S. Silvestro 12, 56127 Pisa, Italy

**Keywords:** polymerization, coarse-grained modeling, reactive molecular dynamics

## Abstract

The radical polymerization process of acrylate compounds is, nowadays, numerically investigated using classical force fields and reactive molecular dynamics, with the aim to probe the gel-point transition as a function of the initial radical concentration. In the present paper, the gel-point transition of the 1,6-hexanediol dimethacrylate (HDDMA) is investigated by a coarser force field which grants a reduction in the computational costs, thereby allowing the simulation of larger system sizes and smaller radical concentrations. Hence, the polymerization is investigated using reactive classical molecular dynamics combined with a dynamical approach of the nonequilibrium molecular dynamics (D-NEMD). The network structures in the polymerization process are probed by cluster analysis tools, and the results are critically compared with the similar all-atom system, showing a good agreement.

## 1. Introduction

Understanding polymerization processes is a challenging and important task because they are widely used in technological applications, such as coating [1,2], 3D printing [3,4], or bioscaffold fabrication [5,6]. The chemical mechanism underlying photopolymerization is free-radical polymerization, see, for example, [7,8], and consists of three steps: (1) initiation; (2) propagation; and (3) termination. Indicating with *R* a radical, with the • an activated radical, and with $M,M_{n}$, respectively, a monomer and a polymer composed of *n* monomers, the three steps take the following form:

(1) Initiation:
  $$\begin{array}{c} R+h\nu =R^{•}\\ R^{•}+M=RM^{•} \end{array}$$
(2) Propagation:
  $$\begin{array}{c} RM_{n}^{•}+M=RM_{n+1}^{•} \end{array}$$
(3) Termination:
  $$\begin{array}{c} RM_{n}^{•}+RM_{m}^{•}=RM_{n+m}R\\ RM_{n}^{•}+RM_{m}^{•}=RM_{n}+RM_{m} \end{array}$$

The first one of the termination reactions is called combination, while the second is disproportionation. Disproportionation is usually less probable than combination at a low-temperature [9]. An amount of radicals is present in the initial polymer solution; the light activates the radicals, which can react and activate the other vinyl groups, converting the carbon double bonds. Only activated groups can react with the unreacted monomers, and this process is called chain-growth polymerization. As the polymerization proceeds, the number of converted double bonds increases. Thus, the percentage of the double bond conversion is typically used as a macroscopic observable to monitor the polymerization process. The complex mechanisms involved in these processes need to be modeled in order to study large systems and time scales at an affordable computational cost.

In a previous work [10], we investigated the structural and dynamical properties of the 1,6-hexanediol dimethacrylate polymerization process using classical all-atom (AA) force fields and reactive molecular dynamics. In Ref. [10], we used the TraPPE classical force field [11,12,13,14,15,16] in the UA atoms version where only the hydrogen atoms are not considered explicitly, but their effect is included in the force field. The remaining atoms, excluding the hydrogen ones, are instead explicitly treated, increasing the system’s degrees of freedom. However, as observed in [10], the network structures of the polymerized systems, such as the number of cycles of a given size (cycles distribution), degree of heterogeneity, volume shrinkage, and Young’s elastic modulus, are insensitive to the initial reactive monomer concentrations, so a simpler description of the monomers fluid may be sufficient. Based on the above, in this paper, we build a coarser force field based on the iterative Boltzmann inversion (IBI) scheme [17,18,19] to understand what aspects of polymerization can be captured by a simpler and computationally more efficient model. Further, we are interested in investigating the capability of the IBI approach to describe not only static characteristics but also dynamical processes, such as radical polymerization. In particular, we are interested in the capability of this coarser model to reproduce two aspects of the polymerization: (1) the gel-point dependence on the initial radical concentration that, as found in [10], occurs at the conversion amount of double bonds that increases when increasing the initial radical concentration and (2) the final network structures. The coarser force field was obtained with the IBI scheme, as will be described in the next section, starting from the TraPPE-UA atoms adopted in [10]. This choice is unnecessary, and a coarser force field can also be obtained starting directly from ab initio simulations of the equilibrium liquid system, without resorting to an existing classical force field. Reducing the number of degrees of freedom in the coarser force field permits to explore larger system sizes and longer time scales [20]. Simulating the system for longer times allows the investigation of low initial radical concentrations, reaching closer to the experimental conditions [21,22,23]. In particular, such advantages make this model useful when one wants to study a problem at large length and time scales where full-atomistic approaches are too expensive, or in-lattice coarse-grained models [20] are too coarse in describing the polymerization process.

Finally, we highlight the use of a topological reactive approach [24] where a topology matching algorithm looks for the specific topology describing the pre-reacted template to add the proper new bonds, once certain conditions are satisfied, mainly the distance reaction cut-off and probability transition coefficients [25]. In the present work, we will also provide the theoretical background of the dynamical approach to nonequilibrium molecular dynamics (D-NEMD) introduced by Orlandini, Meloni, and Ciccotti [26]. The D-NEMD is here used as a theoretical framework to statistically analyze the set of reacting/polymerizing independent trajectories.

The paper is organized as follows: In Section 2, the IBI method and the D-NEMD approach are described alongside coarse and fine model systems. In Section 3, the obtained results are exposed and compared to the findings of [10]. Finally, in Section 4, the conclusions are given.

## 2. Models and Methods

### 2.1. Coarse-Grained Potential

As a starting point, we adopt the description of the 1,6-hexanediol dimethacrylate given by the TraPPE force fields in its united atoms formulation, which does not treat the hydrogen atoms explicitly. Thus, in the TraPPE description, the 1,6-hexanediol dimethacrylate molecule is described by 18 particles. The simulations carried out with the TraPPE force fields in Ref. [10] showed that the final topological and mechanical structures of the systems are independent on the initial radical concentrations while differences in the gel-point location were found. The small dependence on the considered initial parameters suggests that also a coarser model potential, computationally less expansive, might be able to reproduce the same phenomenology. In fact, especially for small initial radical concentrations requiring large system sizes, the simulation can be quite expensive in computational costs. Different approaches are possible to obtain coarse-grained potentials, but we used the IBI option based on the match of the radial distribution functions [17,18,19]. To construct a coarse-grained (CG) potential in the IBI approach, a long trajectory of a more refined model is needed to represent/sample the several degrees of freedom of the system under investigation at the equilibrium state. This trajectory can be obtained from either an ab initio simulation or a classical model potential. In this work, we started from the TraPPE-UA potential to have a direct comparison with the results given in [10] and also to dispense with the high computational costs of ab initio simulations.

**Figure 1:**
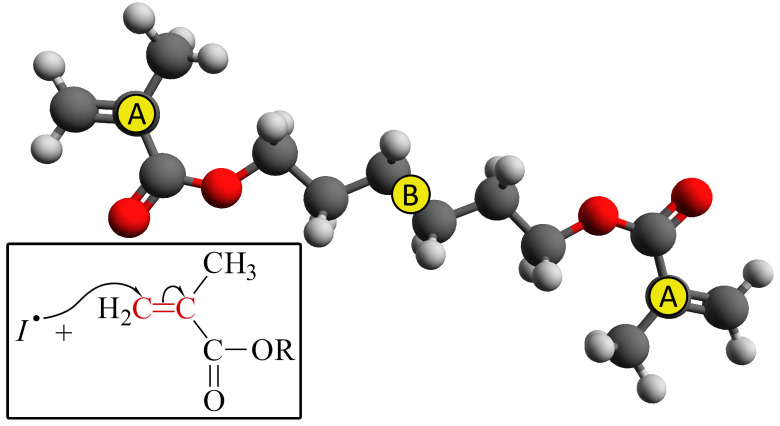
Structure of the 1,6-hexanediol dimethacrylate monomer. With yellow circles, the carbon atoms, of type A and B, used in the CG procedure as described in the text are marked.

To build the CG potentials, we selected 2 carbon atoms and the position of the center of mass of the 1,6-hexanediol dimethacrylate monomer, in Figure 1 marked with yellow circles. The three particles are collected in two types, labeled A and B. The coordinates of the two type A particles together with the type B particle represent the coarser system. This choice of coarser degrees of freedom delivers a factor six between the number of atoms of the coarse-grained (CG) and all-atoms (AA) systems. It is worth noting that there is no mass scale factor between AA and CG systems. In particular, the mass of the GC sites was chosen as the sum of the mass of AA particles building the CG sites: in atomic mass units, 85 u for the A sites and 84 u for the B ones.

The IBI method [17,18,19,27] is based on the seminal work of R. Henderson [28] which establishes the existence of a unique pair potential matching the desired distribution functions. In this case, we have 5 distribution functions divided into short- and long-range types.

(1) $$\begin{array}{c} P^{\theta }\left({\hat{r}}_{AB}\cdot {\hat{r}}_{BA^{\prime }}\right),\;\;P^{bond}\left(r_{AB}\right),\\ g^{AA}\left(r_{AA}\right),\;\;g^{AB}\left(r_{AB}\right),\;\;g^{BB}\left(r_{BB}\right) \end{array}$$

where the P(r) functions are probability densities representing the short-range interactions while the g(r) are radial pair distribution functions used for long-range potentials.

Although in complex systems the IBI could also be applied on more complex correlation functions [29], here we consider only the pair distribution functions. Thus, the corresponding pair interaction potentials are given by:

(2) $$\begin{array}{c} v\left(r_{ij}\right)=-k_{B}T\ln\left[P\left(r_{ij}\right)\right]\\ v\left(r_{ij}\right)=-k_{B}T\ln\left[g\left(r_{ij}\right)\right] \end{array}$$

**Figure 2:**
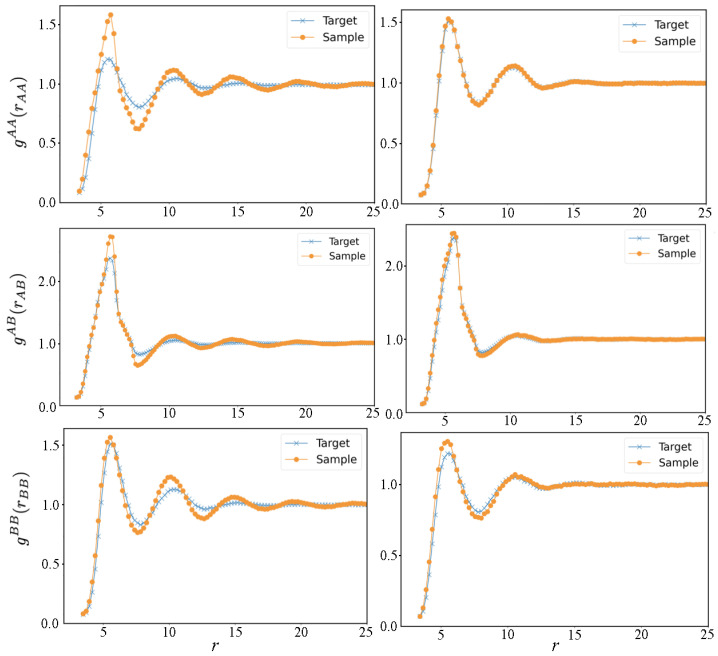
Long-range radial distributions obtained in all-atoms simulations, compared to the coarse-grained ones (distance in Å). In the left panels, the coarse-grained distributions in the first step of the IBI procedure are reported, and the right panels report the distributions in the final iteration.

**Figure 3:**
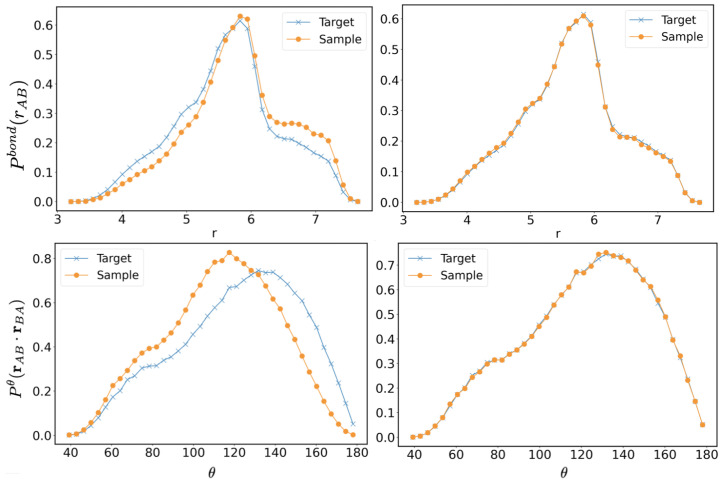
Short-range distributions obtained in AA simulation compared to CG ones (distance in Å). In the left panels, the coarse-grained distributions in the first step of the IBI procedure are reported, and in the right panels, the distributions in the final iteration.

Each interaction potential, at each iteration of the algorithm, is updated as follows:

(3) $$\begin{array}{c} v^{i+1}\left(r_{ij}\right)=v^{i}\left(r_{ij}\right)+\epsilon \ln\left[\frac{P^{i}\left(r_{ij}\right)}{P_{tgt}\left(r_{ij}\right)}\right]\\ v^{i+1}\left(r_{ij}\right)=v^{i}\left(r_{ij}\right)+\epsilon \ln\left[\frac{g^{i}\left(r_{ij}\right)}{g_{tgt}\left(r_{ij}\right)}\right] \end{array}$$

where the distributions with the subscript tgt are the target distributions obtained in the AA system and the parameter ϵ<1 is numerically adjusted to reach the desired convergence. The distance range, $r_{ij}$, considered to generate the CG potential, was taken into the range from zero up to the interaction cut-off value, $r_{\mathrm{cut}-\mathrm{off}}=25$ Å, to account the long-correlation effects. The CG potential at distance larger than $r_{\mathrm{cut}-\mathrm{off}}$ is assumed equal to zero by construction. The rules in Equation (3) simply state to decrease the interaction potential (so that the distribution will be hopefully increased in the successive step) if the obtained distribution $P^{i}\left(r_{ij}\right)$ (or $g_{tgt}\left(r_{ij}\right)$) is lower than $P_{tgt}\left(r_{ij}\right)$ (or $g_{tgt}\left(r_{ij}\right)$) and to increase the potentials in the opposite case. The initial guess of the potentials are obtained using Equation (2) and the target quantities $P_{tgt}\left(r_{ij}\right)$ or $g_{tgt}\left(r_{ij}\right)$. The target (this is needed only in the initial step of the algorithm) and CG distributions were sampled by an NVT ensemble (5 ns trajectory) at 400 K, chosen as the typical operating temperature of the photopolymerization in 3D-printing process, simulated using the Nose–Hoover chain dynamics as implemented in Lammps [30]. The system size of the AA and CG atoms were, respectively, of 6912 and 1152 atoms corresponding to a box length of about 65 Å, the timestep was set to 1 fs for both CG and AA system. In Figure 2 and Figure 3, the initial and final distribution functions compared with the targets are reported. A total of 28 steps of IBI update rules given in Equation (3) were performed, and the parameter ϵ was set between 0 (at some point of the iteration steps, the update of short-range potentials is no longer needed) and 0.4, depending on the iteration step and interaction type. The accordance is very good considering the moderate computation cost: a trajectory of the CG system is calculated in less than one hour using 8 CPU cores.

Once the CG potentials in the NVT ensemble were obtained, we verified that they reproduce the correct density in the NPT ensemble for a larger system composed of 6912 CG atoms and with a box length of about 100 Å (same size of the system in Ref. [10]). Using an NPT Nose–Hoover chain thermostat, we found, at 400 K and 1 atm, a density ρ= 0.893(3) gr/cm${}^{3}$, to be compared to 0.905(2) gr/cm${}^{3}$ in the AA system, which shows a good agreement with only 1% of deviation.

It is worth stressing that the classical reactive molecular dynamics approach hinders other approaches, such as real-time series and maximum likelihood [31], for parametrizing coarse-graining potentials of nonequilibrium extended systems in the actual context due to the high computational costs to achieve the convergence by such schemes. However, the CG potential obtained by the IBI approach was validated a posteriori on dynamical properties, such as the gel-point transition (see Section 3).

### 2.2. Coarse-Grained Reaction Modeling

In the present work, a specific quantity of active monomers was added to model the initiation step. In particular, the number of active radicals reflects the intensity and exposure time of the light irradiating the material. In the literature, photo-activation has been modeled following two different strategies. The first treats the photo-activation as a probabilistic event [32], while the second mimics the effect of a very short intense light beam by setting an initial fixed number of free radicals [33]. Here, we exploit the second strategy, where a set of coarse-grained particles representing the vinyl group among the HDDMA molecules is randomly extracted. Hence, the selected coarse-grained particles are set as radical sites in the initial system, while the other coarse-grained particles representing the vinyl group of the 1,6-hexanediol dimethacrylate were set as potentially reactive atoms. The initial radical concentration is fixed by tuning the ratio between the number of activated radical groups and the total number of vinyl groups.

**Figure 4:**
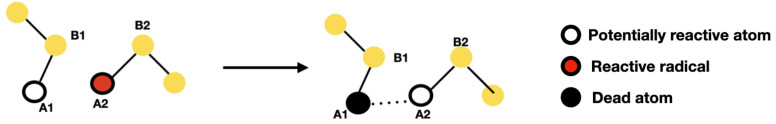
Illustration of the chain propagation reaction in the CG model. A new bond is created between the atoms A1, A2 if a reaction occurs (see text) and two angle potentials, B1-A1-A2 and A1-A2-B2, are added. In red, the reactive atoms; in white, the potentially reactive atoms; and in black, the inactive ones are drawn.

The classical reactive molecular dynamics is used to model the evolution of the double bond conversion occurring in the propagation step. In particular, a topological approach is usually exploited to describe the breaking of the double bond and the consequent formation of a new covalent bond [24,25]. In the LAMMPS code [30], two different topological reactive approaches are available. In the first, pre- and post-templates provide all the topological information, such as type, bonds, angles, dihedrals, of the molecular structures before and after the covalent bond formation between reactive atoms by the bond/react fix package [25]. In the second approach implemented in the bond/create package, a new bond is added between pairs of reacting atoms whenever a specified criterium is verified. In particular, whenever a reactive carbon is below a prescribed distance from a reactive vinyl group (-CH=CH2), the algorithm proceeds to create a new covalent bond (see Figure 4). Consequently, the carbon atoms change type due to the double bond conversion, and a new set of topological information is inserted, including the new bond between the carbon and the vinyl group and the two new angles beside the radical site. In the following, we use the bond/create package implemented in the LAMMPS code [30]. Hence, whenever a radical atom and a potentially reactive carbon atom get closer than a distance threshold, the reaction occurs, and the radical site is transferred to the reacted monomer. Then, the last reacted radical site becomes a dead site after the radical transfer, and the force field interactions of the system need to be updated according to the new topology (newly added bond). Note that in the following, we will refer to the distance threshold as the reactive cut-off distance different from the interaction cut-off, $r_{\mathrm{cut}-\mathrm{off}}$, previously defined. Further, it is worth highlighting that the topology matching algorithm with the CG system description requires less detailed templates to describe the pre- and post-reacted topology, as compared to the AA description, thereby reducing the computational costs of the topology identification step.

The changes in the topology of the systems during the polymerization reaction are illustrated in Figure 4. In particular, referring to Figure 4, when a reactive atom gets closer to the reactive cut-off distance to a potentially reactive atom, a new bond is formed between A1 and A2, and two angles potentials, B1-A1-A2 and B1-A2-B2, are added. Note that the angles potentials were calibrated by fitting within the IBI approach a quadratic (harmonic) function to the probability distribution function of the angle formed by the three centers of mass formed by the three groups of atoms in the AA simulations, representing the corresponding atoms A and B in the coarse-grained representation.

It is worth noting that the CG description usually provides an acceleration in the time evolution of the system given the reduced degree of freedom in the molecular representation [34]. However, we highlight that the slow step in the radical polymerization is mainly due to the double bond conversion, so the possible acceleration due to the CG representation can be neglected. Indeed, the double bond conversion involves an activation free-energy which was theoretically assessed by ab initio calculation equal to 12 kcal/mol at 400 Kelvin [10]. Consequently, even if two monomers are in the configuration to react, following the Eyring equation, it is possible to argue that the probability of observing a double bond conversion is around $10^{-8}$ per femtosecond [10]. Thus, the reaction of polymerization is improbable to occur. For instance, the probability of reacting would be lower than 2% in 1 ns of simulation. In the actual investigation, the probability coefficient in the bond/create package was set equal to 1, so we accelerated the reaction rate by using the reactive strategy in the LAMMPS code. In simple words, a new bond is always added whenever the two reacting atoms are below the prescribed threshold distance. The acceleration above provides a dominant effect, undoubtedly more significant than the acceleration due to the lower degree of freedom in the CG representation. Nonetheless, already in the AA representation, it was observed by Monteferrante et al. [10] that such acceleration does not provide a significant alteration neither in the location of the gelation point nor in the distribution of loop size. Consequently, the argument proves heuristically that the lower degree of freedom in the CG description does not affect essential observables of the polymerization process, such as the gelation point or the polymeric network topology.

### 2.3. Dynamical Approach to Nonequilibrium Molecular Dynamics

To characterize the polymerization process statistically, we exploit the D-NEMD introduced by Orlandini, Meloni, and Ciccotti [26]. In particular, the D-NEMD allows the dynamical investigation of the double bond conversion in the polymerization process and the assessment of the gel point. Following the insights reported by Ciccotti and co-workers [35,36], the D-NEMD exploits an initial state/probability distribution defined by a macroscopic condition (e.g., NVT or NPT equilibrium). Let us indicate $\hat{O}\left(\Gamma \right)$ as a generic observable which is a function of the point in the phase space $\Gamma =(\vec{r},\vec{p})$. The expectation value of $\hat{O}\left(t\right)$ at time *t* along the time evolution of a nonequilibrium system can be estimated as the ensemble average over the probability density function (PDF) f(Γ,t) at time *t*, given as:

(4) $$\begin{array}{c} \bar{O}\left(t\right)=\int_{\Gamma }\hat{O}\left(\Gamma \right)f\left(\Gamma ,t\right)d\Gamma . \end{array}$$

**Figure 5:**
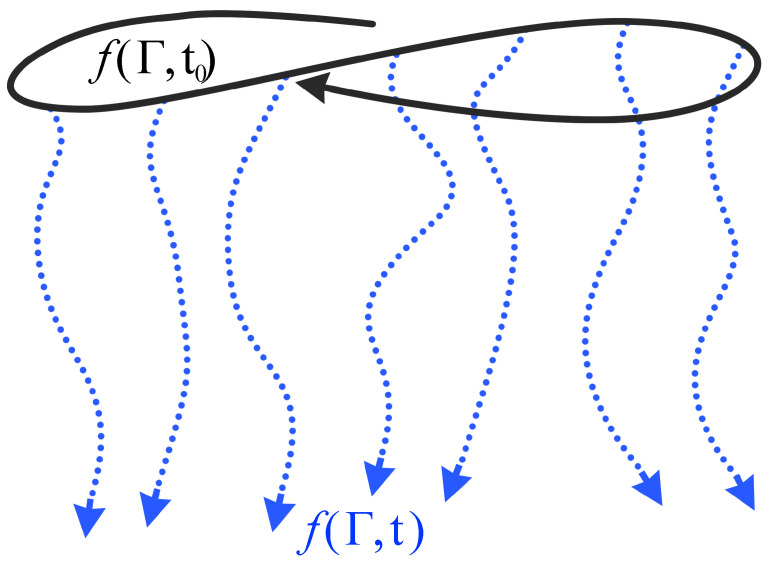
Sketch representing the NEMD paradigm. The continuous line represents the trajectory sampling the initial PDF $f\left(\Gamma ,t_{0}\right)$, at the initial time, $t_{0}$. The pointed lines represent the independent trajectories evolved in time up to *t* sampling $f\left(\Gamma ,t\right)$ from the initial configurations belonging to $f\left(\Gamma ,t_{0}\right)$.

Note that f(Γ,t) respects the Liouville equation:

(5) $$\begin{array}{c} \frac{\partial f}{\partial t}=-\left\{f\left(\Gamma ,t\right)\hat{H}\left(\Gamma ,t\right)\right\}=-iL\left(t\right)f\left(\Gamma ,t\right), \end{array}$$

where $L\left(t\right)$ is the Liouville operator while $\left\{\cdot ,\cdot \right\}$ denotes the Poisson bracket. The D-NEMD allows to get around the direct evaluation of the PDF, $\Gamma =(\vec{r},\vec{p})$ at time *t*, which is extremely difficult to be obtained, apart from simple cases. Indeed, the D-NEMD exploits the Onsager–Kubo relation:

(6) $$\begin{array}{c} \bar{O}\left(t\right)=\int_{\Gamma }\hat{O}\left(\Gamma \right)S^{*}\left(t\right)f\left(\Gamma ,t_{0}\right)d\Gamma =\int_{\Gamma }S\left(t\right)\hat{O}\left(\Gamma \right)\left(t\right)f\left(\Gamma ,t_{0}\right)d\Gamma , \end{array}$$

denoting $S\left(t\right)$ the time-evolution operator of Γ, and $S^{*}\left(t\right)$ its adjoint, representing the time-evolution operator of the PDF. However, whenever the initial system at initial time $t_{0}$ is macroscopically determined (e.g., given temperature and pressure), the PDF, $f\left(\Gamma ,t_{0}\right)$, at the initial time, $t_{0}$, can be easily sampled because it is described by a conditional probability distribution. Thus, Equation (6) can be utilized to compute $\bar{O}\left(t\right)$ without the necessity to compute $f\left(\Gamma ,t\right)=S^{*}\left(t\right)f\left(\Gamma ,t_{0}\right)$, but simply using the set of $\hat{O}\left(t\right)$ values evolved up to time *t* along *N* independent trajectories started from a set of phase-space points, ${\left\{\Gamma_{0}^{i}\right\}}_{i=1,N}$ distributed over the conditional probability distribution PDF $f\left(\Gamma ,t_{0}\right)$ at time $t_{0}$. Hence, at any time *t*, the time-dependent average $\bar{O}\left(t\right)$ is assessed as:

(7) $$\begin{array}{c} \bar{O}\left(t\right)=\frac{1}{N}\sum_{i=1}^{N}\hat{O}\left(\Gamma (t,\Gamma_{0}^{i})\right), \end{array}$$

where $\hat{O}\left(\Gamma (t,\Gamma_{0}^{i})\right)$ denotes the value of the observable, $\hat{O}$, assumed at time *t* along the *i*-th trajectory started from the phase-space point, $\Gamma_{0}^{i}$ (see sketch in Figure 5). In the present work, we assume that $f\left(\Gamma ,t_{0}\right)$ is the PDF of a system at a constant number of atoms, temperature, and volume without any double bond conversion.

## 3. Results and Discussion

For a direct comparison between the CG and AA descriptions, we select three different initial radical concentrations, namely 1%,3%, and 5%. In all the three cases, the initial system contains 2250 monomers of HDDMA. The use of an NPT ensemble to simulate the polymerization process is not possible because the CG potential is generally not transferable to other thermodynamic conditions, such as density. However, complex correction terms can be added to the CG force field to allow thermodynamics transferability. A notable example is the embedded atom model [37] and approaches involving the extra terms accounting for two, three, and n-body particle interactions [38]. Here, we limit the investigation to the NVT ensemble, avoiding using additional terms in the force field, to be explored in future work.

**Figure 6:**
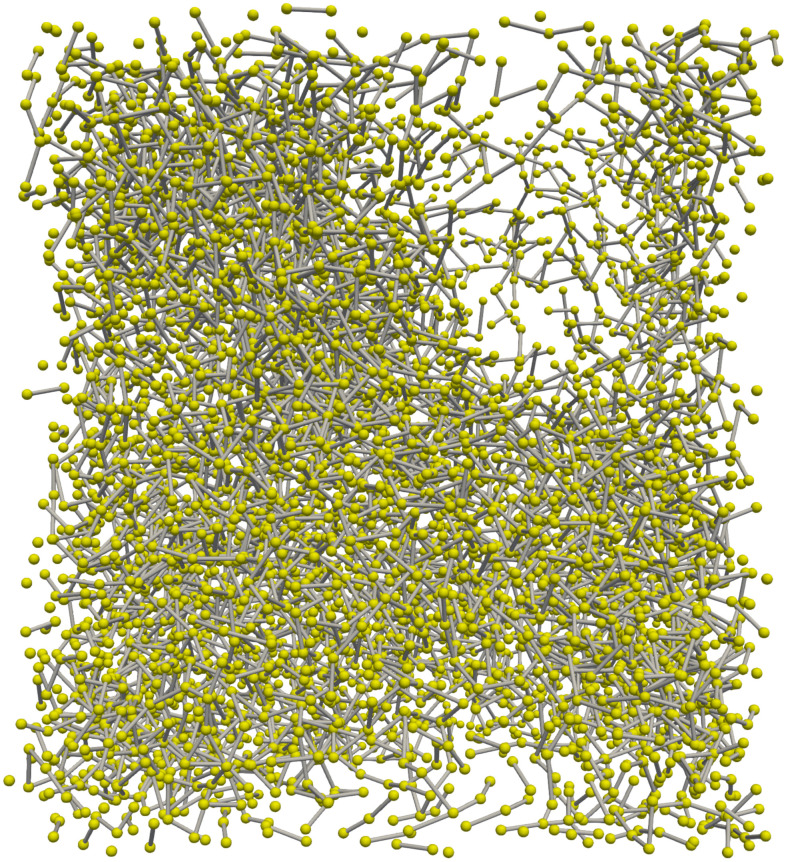
Snapshot of the polymeric structure with an initial radical concentration of 3% and a 40% double bonds conversion without the pressure correction term. Note that the bonds crossing the periodic boundaries are not drawn in the figure.

At first, we chose Langevin dynamics to sample the NVT ensemble, so that by adjusting the damping constant (usually the inverse indicated with γ) of the thermostat, the diffusion coefficient was tuned in order to match the self-diffusion coefficient of the HDDMA monomer. In particular, we used (1/γ)= 57 fs and T = 400 K, the same temperature at which the CG potential was built, obtaining a self-diffusion coefficient D = (1.18±0.5)×10${}^{-5}$ cm${}^{2}$/s, which is very close to 1.17×10${}^{-5}$ cm${}^{2}$/s obtained for the AA case [10]. It is worth highlighting that the self-diffusion coefficient of HDDMA was not experimentally assessed. Nonetheless, the actual self-diffusion coefficient is in good agreement with the value experimentally observed for the hydroxyethyl methacrylate dimer [39] which is about $0.98\times 10{}^{-5}$ cm${}^{2}$/s at 323 K. The reactive cut-off distance for the CG force field at which the reaction takes place has been chosen in the following way: starting from an initial reaction cut-off equal to the value assumed in the AA system, we simulate the system considering the 3% concentration and adjust the reaction cut-off so that in the first 20 ps of polymerization, the difference in the double bond conversion reached by the CG and AA falls below 1%. Note that the reaction cut-off in the AA system was set to 4 Å as in the previous works [10,33]. The extension of the potential obtained in the monomer liquid to the polymerized system gives rise to pathologies of the polymerization process: for all concentrations, we observed inhomogeneity of the system density with small unoccupied empty regions, as evident in Figure 6. This behavior also appears in the first stages of polymerization. For example, Figure 6 refers to 40% double bonds conversion, but it is visible also at early stages and causes a slowdown of the monomer mobilities and polymerization velocity. The system pressure decrease represents a clear manifestation of non-transferability as the reaction proceeds. In the NVT ensemble, the pressure is not imposed by the thermostat and can be calculated using the virial:

(8) $$\begin{array}{c} P=\frac{Nk_{B}T}{V}+\frac{\sum_{i=1}^{N}{\vec{r}}_{i}\cdot {\vec{F}}_{i}}{3V} \end{array}$$

where *N* is the number of atoms, $k_{B}$ the Boltzmann constant, *T* the temperature, ${\vec{r}}_{i}$ the position of the atom *i*, and ${\vec{F}}_{i}$ the total force acting on the atom *i*. In the 5% of the initial radical concentration and at 50% of the double bonds conversion, the pressure was decreased down to −500 atm, largely lower than the target pressure of −35(± 1) atm obtained in a corresponding polymerization simulation in an NVT ensemble with the AA force field.

As aforementioned, it is possible to obtain more general potentials, for example, with pressure or density transferability, by adding extra terms accounting for two, three, and n-body particle interactions at the cost of a more complex form of the CG potential [38]. In the present work, we follow a different and simpler approach. At 50% of the double bond conversion, which is easily reached by all the considered concentrations, we correct the long-range potential $g^{AA}\left(r_{AA}\right)$ using the ramp correction introduced by G. Milano and F. Müller-Plathe in Ref. [40] to adjust the pressure:

(9) $$\Delta v^{AA}\left(r_{AA}\right)=\Upsilon_{0}\left(1-\frac{r_{AA}}{r_{\mathrm{cut}-\mathrm{off}}}\right).$$

**Figure 7:**
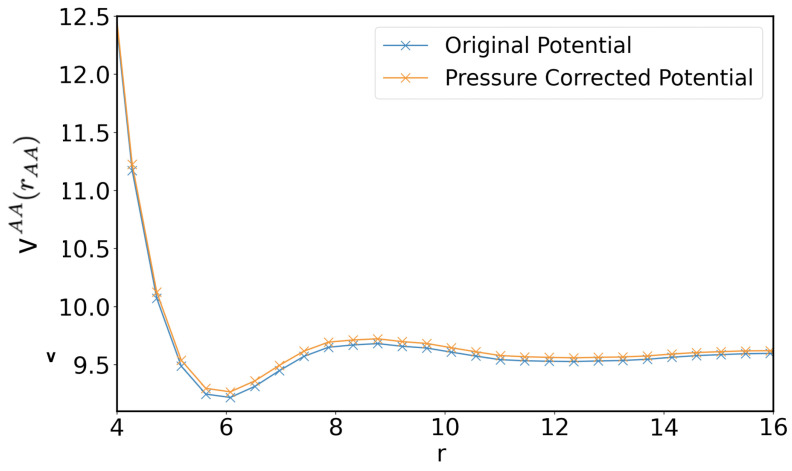
Comparison of the original coarse-grained potential in Kcal/mol obtained with the IBI method and the pressure corrected one for the inter-particle term AA as a function of their mutual distance in Å.

Pressure corrections can be, in general, used in the IBI scheme whenever the CG potential shows a pressure discrepancy from the target value of the AA system [40]. Equation (9) adds a weak linear potential term $\Delta v^{AA}$ to the attractive long-range part of the potential $v^{AA}$ obtained from the iterative procedure of Equation (3). Note that the correction term is applied as an a posteriori correction applied to the force field obtained from the IBI procedure. Thus, the parameter $\Upsilon_{0}$ in Equation (9) is adjusted to obtain a pressure of the polymerized CG system close to the target pressure, keeping the interaction cut-off value $r_{\mathrm{cut}-\mathrm{off}}=25$Å. Note that the correction parameter $\Upsilon_{0}$ is negative or positive depending on the pressure, which can be above or below the target value. In our case, with $\Upsilon_{0}=0.02$ kcal/mol, we obtained a scaled form of the CG force field, of which the potential function of the term AA is reported as an example in Figure 7. In the polymerization process, whenever a CG atom is dead, the CG atom changes type, and its interaction potential with all other atoms of type A is changed to $v^{AA}+\Delta v^{AA}$. Using this simple strategy, we were able to avoid the density inhomogeneity in the polymerized system while maintaining the pressure values until about 80% of the double bonds correction, much more close to the target system pressure measured in the AA system: respectively, for the 1%,3%, and 5% cases, we obtained a pressure of −9(± 1), −15(± 2), and −11(± 2) atm for the CG-corrected force field. However, in the CG simulations, we still observe a deviation of about 20% in the volume shrinkage, indicating a pathological behavior that could likely be corrected using more complex force fields, such as the embedded-atom method potentials proposed by Daw and Baskes [37,41].

**Figure 8:**
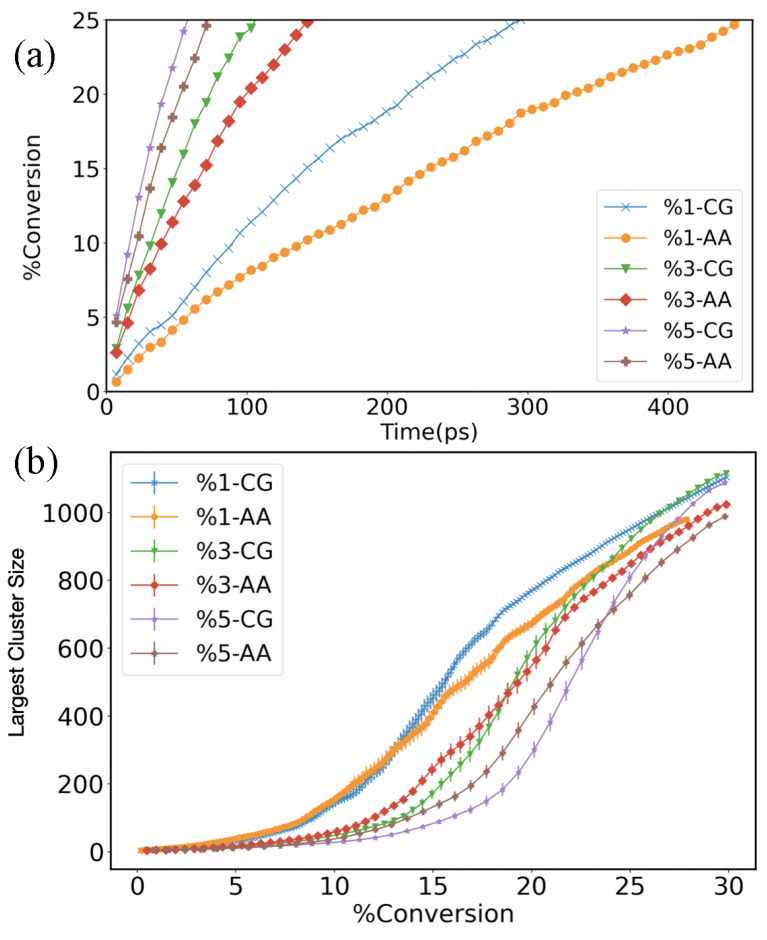
(**a**) Double bond conversion as a function of simulation time averaged by D-NEMD for the full-atomistic (AA) and the coarse-grained (CG) force fields at different initial radical concentrations. (**b**) Largest cluster size in function of double bond conversion. The curves were used to calculate the gel points.

To calculate the gel point, we performed 20 runs for each concentration for a sufficient time to reach 30% of the double bond conversion. In particular, by Equation (7), we computed the double bond conversion as a function of time *t* averaged over the set of 20 independent trajectories, exploiting the D-NEMD (see Figure 8). Hence, the gel point was calculated fitting the curve representing the largest cluster size assessed by the cluster analysis tools (Depth-first search) as a function of the conversion of the double bonds with the following fitting expression [10,33,42]:

(10) $$n_{max}\left(x\right)=a-\frac{a}{\left[1+{\left(\frac{x}{b}\right)}^{c}\right]}+d\;x$$

where a,c,b,d are the fitting parameters and the gel point $x_{gel}=b{\left[\left(c-1\right)/\left(c+1\right)\right]}^{1/c}$. The curves representing the largest cluster versus the radical conversion are shown in the bottom panel of Figure 8 and the values of the gel points for the CG system are $x_{gel}^{CG}≃\left\{15.1\pm 0.3,19.5\pm 0.4,22.6\pm 0.3\right\}$, respectively, for the 1%,3%, and 5% initial reactive radical concentration. In the AA force field systems, we obtained $x_{gel}^{AA}≃\left\{15.8\pm 0.9,18.9\pm 0.4,21.2\pm 0.4\right\}$, respectively, which are in good agreement with the corresponding values in the CG simulations.

**Figure 9:**
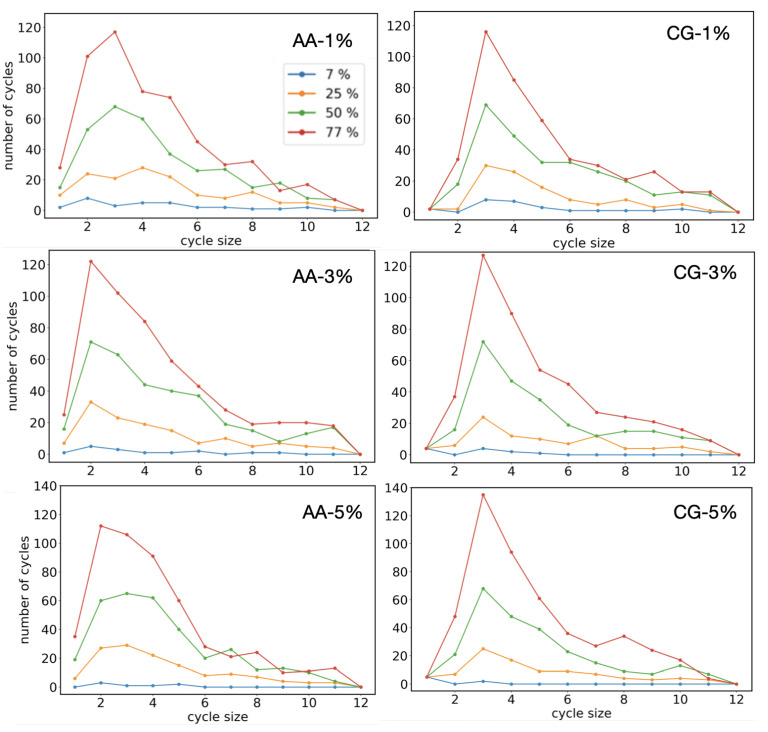
Cycles size distribution for the AA (from Ref. [10]) and CG systems at different stages of the polymerization process in double bonds conversion values and different initial radical concentrations. The distribution was computed as the mean over a set of 20 independent trajectories. The standard deviation (not reported in figure) was found to be always less than 5% of the corresponding mean values.

The cycles size distributions of the CG and AA model are also reported in Figure 9 for different values of the initial reactive radical concentration to characterize the final network structures. In Figure 9, the difference in the cycle distribution of sizes one and two in all the concentrations is clear: a smaller number of cycles with sizes one and two is present in the AA model compared to the CG simulations in all the reaction stages. This result is not surprising because the coarse-grained angle potential was not modified during the polymerization. Consequently, the angle formed from reacted monomers is too stiff to allow the formation of cycles with sizes one and two during the polymerization process. On the contrary, the cycles size distributions for sizes larger than two show good agreement.

Finally, we report a speed-up factor of about eight times in the wall-clock time using the CG approach compared to the standard AA description for a system containing the same number of initial monomers, which likely allows the investigation of a larger system in monomer quantity at the same computational costs. The speed up is mainly due to the lower number of particles to describe each monomer, granting a significant reduction in the computational costs of the N− body problem related to the assessment of the long-range interaction term, usually scaling as (NlogN).

## 4. Conclusions

In general, CG potentials are not transferable to different thermodynamics conditions from the ones they were generated for. Moreover, there is no guarantee that dynamical properties are reproduced. Further, it is worth highlighting that CG potentials suffer limitations due to the inverse inferential procedure of the IBI scheme, where the system’s underlying properties are inferred by the system’s physical properties (as the radial distribution function). In other words, the practical use of Henderson’s theorem [28] is limited. The theorem states that two potential energy functions produce the same radial distribution function only if the potential functions differ by a constant. On the other hand, small input data changes may lead to dramatically different results in the inverse inferential procedure [43]. To overcome the issue, we investigated the structural and dynamical properties of the 1,6-hexanediol dimethacrylate polymerization process, such as the gel-point transition, and several properties of the polymerized systems, such as the number of cycles of a given size, degree of heterogeneity, and volume shrinkage. All these features have been compared with the corresponding data obtained in the all-atomistic simulations to probe the robustness of the assessed CG potentials. Therefore, the practical limits in the iterative Boltzmann inversion scheme have been tested with several physical and topological properties of the system beyond the distributions strictly employed to infer the CG potentials. In particular, by studying the polymerization of 1,6-hexanediol dimethacrylate, we found that the trend of the gel point as a function of the initial radical concentration is well reproduced with a less expensive CG representation. The gel points of the AA and the CG system are very similar (equal in 2σ). At the same time, the network structures are very similar with comparable cycles size distributions, apart from small cycles with sizes one and two. Further, by adjusting the diffusivity of the CG system atoms and the pressure correction factor, we obtained the same gel-point dependence on the initial radical concentration observed in the AA simulations with a simpler and less computationally expensive CG model. In numbers, we measured a speed-up factor of about eight times in the wall-clock time by using the CG approach compared to the standard AA description. Note that the speed up was observed running both the AA and CG simulations by LAMMPS on 32 CPU processes exploiting the Message Passing Interface library with the same number of monomers in the system (an equivalent polymer size). This allows to consider lower values of the initial reactive radical concentrations, closer to the experimental ones, thereby granting the possibility to explore larger system sizes and longer time spans at affordable computational costs and to obtain dynamical information by D-NEMD.

## Data Availability

The complete set of files containing the coarse-grained force fields in the LAMMPS format can be downloaded from the open repository GITHUB at the website (accessed on 25 October 2022): https://github.com/copmat/HDDMA-CG-POT.
